# Targeting of the *Yersinia pestis* F1 capsular antigen by innate-like B1b cells mediates a rapid protective response against bubonic plague

**DOI:** 10.1038/s41541-018-0087-z

**Published:** 2018-10-19

**Authors:** Yinon Levy, Yaron Vagima, Avital Tidhar, Moshe Aftalion, David Gur, Uri Nili, Theodore Chitlaru, Ayelet Zauberman, Emanuelle Mamroud

**Affiliations:** 1 Department of Biochemistry and Molecular Genetics, Israel Institute for Biological Research, P.O. Box 19, Ness-Ziona, 74100 Israel; 2 Department of Pharmacology, Israel Institute for Biological Research, P.O. Box 19, Ness-Ziona, 74100 Israel

## Abstract

The generation of adaptive immunity by vaccination is usually a prolonged process that requires multiple dosing over several months. Hence, vaccines are administered for disease prevention a relatively long time prior to possible infection as opposed to post-exposure prophylaxis, which typically requires rapid intervention such as antibiotic therapy. The emergence of pathogens resistant to common antibiotic treatments has prompted the search for alternative therapeutic strategies. We previously demonstrated that vaccination of mice with the F1 capsular antigen of *Yersinia pestis* elicits specific and effective yet, unexpectedly, rapid anti-plague immunity. Here, we show by applying genetic and immunological approaches that the F1 antigen is targeted by peritoneal innate-like B1b cells that generate a prompt T-independent (TI) anti-F1 humoral response. The rapid F1-mediated defense response was diminished in *Xid* (Btk^m^) mice in which B1 cell numbers and activity are limited. Binding of fluorophore-labeled F1 to peritoneal B1b cells was detected as soon as 6 h post vaccination, emphasizing the high speed of this process. By assessing the ability to achieve rapid immunity with monomerized F1, we show that the natural polymeric structure of F1 is essential for (i) rapid association with peritoneal B1b cells, (ii) early induction of anti-F1 titers and (iii) rapid TI immunity in the mouse model of bubonic plague. These observations shed new light on the potential of novel as well as well-known protective antigens in generating rapid immunity and could be implemented in the rational design of future vaccines.

## Bubonic plague: Mechanisms of a rapid antibody response

Full acquired immunity normally takes weeks to develop, however it’s possible to raise rapid (days) protective antibody responses against the bubonic plague bacterium *Yersinia pestis* by targeting its F1 capsular antigen. Emanuelle Mamroud and colleagues at the Institute of Biological Research in Israel use genetic and functional studies to understand the mechanism of this rapid anti-F1 antigen vaccine response. Subcutaneous vaccination with polymeric F1 antigen within hours results in selective binding to peritoneal B1b cells. Within seven days this generates an antibody response protective against fully virulent *Y. pestis*. This protection is spleen- and T-helper cell-independent but requires B1b cells. The form of F1 is also important, with monomerized antigen resulting in significantly inferior protection. These findings highlight the potential of post-exposure prophylaxis and the insights into its mechanism having wider implications for vaccine development.

## Introduction

Plague is a fatal, rapidly progressing infectious disease initiated by the gram-negative bacterium *Yersinia pestis*. Approximately 200 million people succumbed to this devastating disease in three pandemic outbreaks in the last two millennia.^1^ Plague is currently an endemic in several regions of the world, including central Asia and Africa, where it causes significant morbidity and mortality, WHO 2017.^2^ The island of Madagascar is the most active focus of plague and recently experienced a severe outbreak, WHO Plague-Madagascar.^3,4^ The most prevalent form of this disease in nature is bubonic plague, which is acquired following a bite from an infected flea.^5^ Without prompt effective antibiotic treatment bubonic plague may develop into the highly fatal pneumonic plague.^6^ The recent isolation of virulent *Y. pestis* strains, which are resistant to several antibiotics including those recommended by the Centers for Disease Control and Prevention (CDC) for therapy, highlighted the need for additional countermeasures.^7,8^

The capsular protein of *Y. pestis* (F1) was used in 1952 as a subunit protective antigen in animal models of plague.^9,10^ The F1 protein is encoded from the *caf1* operon, also including the transcriptional regulator Caf1R, the chaperon Caf1M and the usher protein Caf1A.^11–14^ In the generation of the capsule, F1 forms high-molecular-weight homo-polymers that are exported to the surface of the bacteria.^15,16^ Recombinant F1 polymers can be easily purified from *E. coli* cultures expressing the *caf1* operon and upon immunization, F1 affords protection against bubonic and pneumonic plague in animal models.^15,17–19^ A fusion of a monomerized form of F1 with another protective antigen LcrV, a pivotal component of the type III secretion system of *Y. pestis*, comprise the most promising anti-plague subunit vaccine that is under development.^20–25^ The protective mechanism induced by F1 is based on the serological arm of adaptive immunity.^18,26,27^ Passive transfer of anti-F1 antibodies to naive animals and generation of anti-F1 IgG titers following active immunization with F1 lead to high survival rates in animal models of plague.^28^ Vaccination with F1, but not with LcrV, was shown to rapidly induce anti-plague immunity within several days.^29,30^ We have also shown that the protective response, characterized by the rapid induction of specific anti-F1 antibodies, requires the existence of mature B cells.^30^ In this manuscript, we further elucidate the immune mechanisms behind this rapid F1-mediated protective response and demonstrate for the first time that innate-like B1b cells are an important component of this rapid and effective defense mechanism. The implications of these observations for the development of subunit vaccines that would be advantageous for the rapid induction of protective immunity during a sudden outbreak of infectious diseases is further discussed.

## Results

### The *Y. pestis* F1 antigen induces a rapid T-cell-independent protective humoral immune response against bubonic plague

Acquired immunity generated by vaccination is a prolonged process that usually requires weeks to develop. We have previously shown that F1 is capable of generating rapid B-cell-dependent immunity against plague within several days.^30^ The rapid induction of anti-F1 antibodies suggests the involvement of innate-like fast-responding B cells such as B1 cells or marginal zone (MZ) B cells in a T-cell-independent manner.^31,32^ To directly assess this possibility, the contribution of T-helper cells to the F1-mediated rapid immunity against plague was evaluated.

**Fig. 1:**
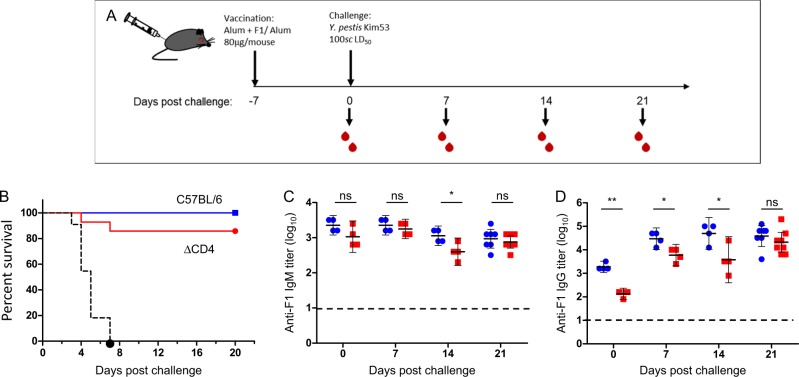
F1 antigen elicits T-helper cell-independent protective immunity. **a** Graphic description of mouse immunization, bleeding and challenge schedule. **b** Survival curves of wild-type C57BL/6J (blue line, *n* = 14) and ΔCD4 mice (red line, *n* = 14) vaccinated with F1 (polymeric form, 80 μg/mouse) 7 days prior to a lethal subcutaneous challenge with the fully virulent *Y. pestis* Kim53 strain (100 LD_50_). Control mice were vaccinated with alum (*n* = 11, dashed black line). Mice were vaccinated as described above. Kinetics of anti-F1 IgM **c** and IgG **d** titer development in the sera of wild-type mice (blue circles) and ΔCD4 mice (red squares) on the day of the challenge (day 0) and every 7 days afterwards up to day 21 post challenge (For both groups, *n* = 4 on all days post challenge except for day 21 on which *n* = 8). Horizontal dashed lines indicate the limit of detection. Short horizontal lines represent the geometric mean titers (GMT ± 95% confidence intervals; CI). (*P < 0.05, **P < 0.005, ns = nonsignificant)

Wild-type C57BL/6J mice and isogenic ΔCD4 mice were immunized with F1 and, 7 days later, challenged subcutaneously with the fully virulent *Y. pestis* Kim53 strain (100 LD_50_). Control mice were vaccinated only with alum and similarly challenged (Fig. 1a). As shown in Fig. 1b, both mouse strains were effectively protected against the challenge, while all control animals succumbed within 7 days (mean time to death = 4 days). While a minor decrease in protection efficiency (90% compared to 100%) was observed in vaccinated ΔCD4 mice, this result indicated, as suggested, that assistance of T-helper cells has a marginal contribution to the F1-mediated rapid protective response

Prior to challenge, comparable anti-F1 IgM titers were generated in the sera of all F1-immunized mice, whereas anti-F1 IgG titers developed by the ΔCD4 mice were lower than those generated by wild-type animals (Fig. 1c, d, day 0). Notably, after the challenge, anti-F1 IgG titers were induced in both mouse strains and reached equivalent levels after 21 days (Fig. 1d). No significant difference was observed between the anti-F1 IgG titers recorded in challenged vs. non-challenged mice, indicating that anti-F1 IgG titers stem mainly from the response to vaccination with F1 (Supplementary Fig. S2). These findings clearly demonstrate that F1 is able to induce a rapid and specific T-cell-independent humoral response that contributes to the survival of the T-helper cell-deficient mice.

### Innate-like B1 cells, but not MZ B cells, are involved in rapid F1-mediated immunity

To identify the B-cell population that mediated the early anti-F1 humoral response, splenectomized mice lacking spleen-restricted MZ B cells were vaccinated with F1 and subcutaneously challenged 3 days later with the *Y. pestis* strain Kim53.

All F1-vaccinated mice survived the challenge, whereas control animals that were vaccinated only with the adjuvant, succumbed to the infection by day five (data not shown). This indicates that MZ B cells had a dispensable role in affording F1-mediated rapid onset of anti-plague immunity.

**Fig. 2:**
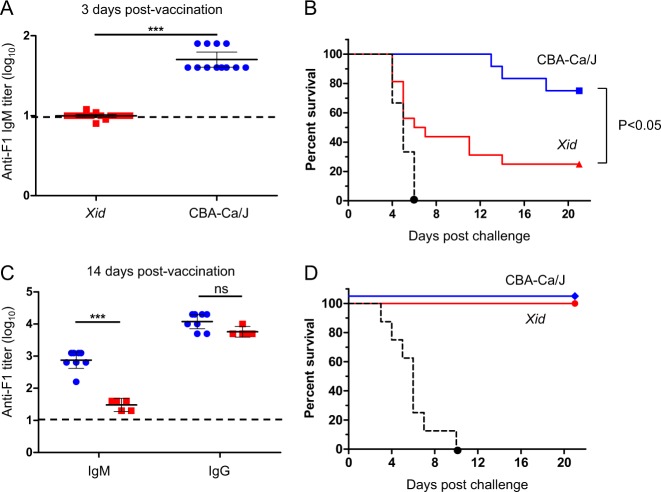
Innate-like B1 cells are involved in the rapid generation of F1-mediated anti-plague immunity. **a** Anti-F1 IgM titers in the sera of wild-type CBA-Ca/J mice (blue circles, *n* = 12) and *Xid* mice (red squares, *n* = 12) 3 days post vaccination with F1 (polymeric form, 80 μg/mouse). **b** Survival curves of CBA-Ca/J (blue line, *n* = 12) and *Xid* mice (red line, *n* = 12) vaccinated with F1 3 days prior to a lethal challenge with *Y. pestis* Kim53. Control mice were vaccinated with alum (*n* = 9, dashed black line). **c** Anti-F1 IgM and IgG titers in the sera of CBA-Ca/J mice (blue circles, *n* = 8) and *Xid* mice (red squares, *n* = 5) 14 days post vaccination with F1. **d** Survival curves of CBA-Ca/J mice (blue line, *n* = 8) and *Xid* mice (red line, *n* = 5) vaccinated with F1 14 days prior to the challenge indicated above. Black dashed line indicates the survival of alum-vaccinated animals (*n* = 8). Short horizontal lines mark the GMT ( ± 95% CI). Horizontal dashed line indicates the limit of detection. (***P < 0.001, ns = nonsignificant)

Next, we examined the contribution of B1 cells to the rapid protective immunity by using CBA-NJ (*Xid*) mice. These mice harbor a specific mutation in the Bruton’s tyrosine kinase gene (*btk*^m^) that results in reduced numbers of B1 cells and an inability to mount serological responses against T-independent (TI) antigens.^33^ Wild-type and *Xid* mice were vaccinated with F1 and, 3 days later, were tail-bled and challenged as indicated above. As expected from the reduced activity of B1 cells in *Xid* mice, anti-F1 IgM titers were not detected in the sera of these mice (Fig. 2a). Accordingly, during the first 12 days after the challenge, all wild-type mice were still protected, whereas 70% of the *Xid* mice were not (Fig. 2b). This result shows that innate-like B1 cells were required for the rapid generation of anti-F1 IgM antibodies as well as for the rapid development of a protective immunity against plague. Twenty-one days after the challenge, a significant difference was observed between the survival rates of the F1-vaccinated wild-type and *Xid* mice (Fig. 2b, *P* < 0.05).

To exclude the possibility that *Xid* mice have a general defect in mounting humoral immune responses against F1, both mouse strains were vaccinated with F1 and challenged with *Y. pestis* 2 weeks later, thereby allowing activation of the slower-developing acquired humoral response against F1. Analysis of anti-F1 titers in the circulation prior to the challenge revealed that *Xid* mice developed much lower anti-F1 IgM titers than those found in wild-type animals, while both mouse strains generated comparable and high anti-F1 IgG titers (Fig. 2c). Consequently, all F1-vaccinated mice were fully protected from the lethal plague infection (Fig. 2d). These results show that *Xid* mice do not have a general defect in inducing a protective humoral immune response against F1 and that the inability to generate the rapid F1-mediated protective response may be attributed to the dysfunctionality of their B1 cells.

### The F1 antigen binds peritoneal B1b cells shortly after immunization

**Fig. 3:**
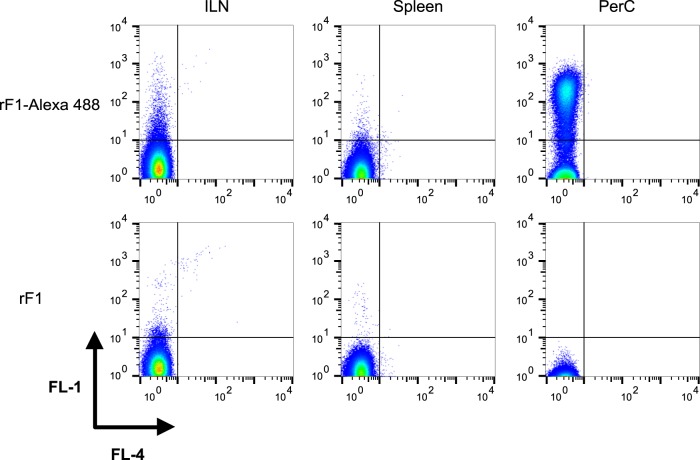
F1 is associated with peritoneal cells shortly after vaccination. Representative dot plot analysis of gated cells isolated from the draining inguinal lymph node (ILN), spleen and peritoneal cavity (PerC) of mice (*n* = 6) vaccinated with labeled F1 (polymeric form, 80 μg/mouse, upper panel). Control mice were vaccinated with non-labeled F1 (*n* = 3, lower panel). FL1 and FL4 indicate fluorescence detected through 530/30 nm and 661/16 nm filters of a FACSCalibur™ flow cytometer (BD Bioscience), respectively. Quadrant lines mark negatively and positively stained cells

**Fig. 4:**
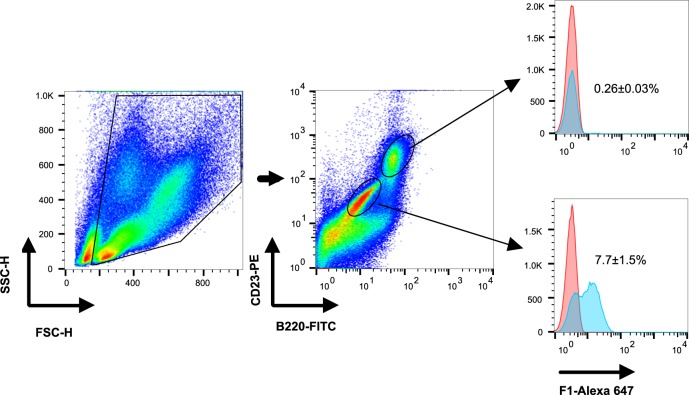
F1 is associated primarily with peritoneal B1 cells shortly after vaccination. Representative dot plot and histogram analyses of gated cells isolated from the peritoneal cavity of C57BL/6J mice injected with alum (red histogram, *n* = 3) or with labeled F1 (polymeric form, 80 μg/mouse, blue histogram, *n* = 6). Isolated cells were stained with markers that identify peritoneal B2 cells (B220^hi^CD23^+^) and B1 cells (B220^mid^CD23^-^). B-cell populations were examined for staining with the labeled F1 (blue histograms) vs. background (red histograms)

**Fig. 5:**
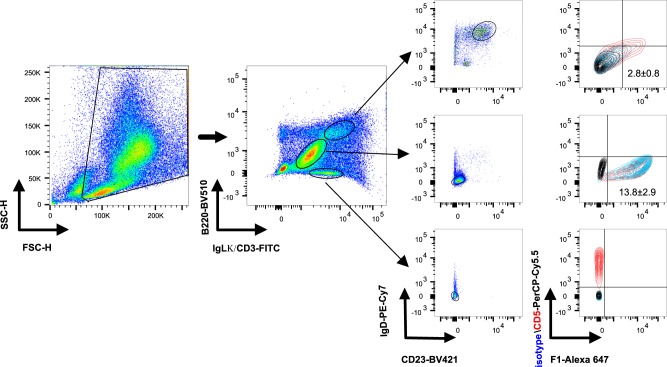
F1 binds peritoneal B1b cells shortly after vaccination. Representative dot plot and contour plot analyses of gated cells isolated from the peritoneal cavity of C57BL/6J mice one day after vaccination with labeled F1 (polymeric form, 80 μg/mouse, *n* = 14 mice). Peritoneal B cells were stained with specific markers for B2 cells (B220^hi^IgLκ^hi^IgD^+^CD23^+^, upper panel) and B1 cells (B220^mid^IgLκ^mid^IgD^-^CD23^-^, middle panel). Labeling of peritoneal T cells (B220^-^CD3^+^IgD^-^CD23^-^, lower panel) was performed as a control, indicating positive staining of the CD5 marker (red contour) and negative staining of the isotype control antibodies (blue contour). Each of the gated cell populations was also examined for staining with labeled F1. Quadrant lines marking CD5 and F1 ‘‘positive’’ populations were determined by plotting the relevant cell population isolated from alum-vaccinated mice (*n* = 4) that was stained with isotype control antibodies (black contour)

To further characterize the interaction between B1 cells and F1, mice were vaccinated with labeled F1. On the next day, cells from lymphatic organs (spleen and the draining inguinal lymph node) and from the peritoneal cavity (PerC) were isolated and analyzed by flow cytometry. Positively stained cells could be detected only in the peritoneal cavity of mice vaccinated with the fluorophore-conjugated F1 (Fig. 3, upper right panel). The specificity of this observation was demonstrated by vaccination with non-labeled F1, which proved that the positive signal did not result from auto-fluorescent cells (Fig. 3, lower panel). Next, we determined which cell population in the peritoneal cavity bound the F1 antigen. Twenty-four hours post vaccination, peritoneal cells were isolated and stained with markers for relevant immune cell populations. As shown in Fig. 4, at this early time point, the majority of the labeled F1 was bound to innate-like B1 cells (B220^mid^CD23^low^). Low yet detectable F1 staining was also identified on follicular B2 cells (Fig. 4, B220^hi^CD23^+^, and Fig. 5 upper panels, B220^hi^IgLκ^hi^IgD^+^CD23^+^CD5^−^). No labeling could be detected on peritoneal polymorphonuclear neutrophils (PMNs), dendritic cells (DCs), NK and T cells (data not shown). Staining of the peritoneal cells with additional B-cell markers revealed that B1b cells were the major F1-binding population (B220^mid^IgLk^mid^IgD^−^CD23^−^CD5^−^, Fig. 5 middle panel, 13.8 ± 2.9% of PerC cells). Staining of peritoneal T cells was used as a positive control for the expression of CD5 (CD3^+^B220^−^IgD^−^CD23^−^CD5^+^, Fig. 5 lower panel). The ability of F1 to elicit an extremely rapid B1b-mediated immune response was further highlighted by the observation that binding could be detected as early as 6 h post vaccination and up to 4 days afterwards (Supplementary Fig. S3).

### The polymeric structure of the F1 antigen is required for the induction of rapid F1-mediated immunity

**Fig. 6:**
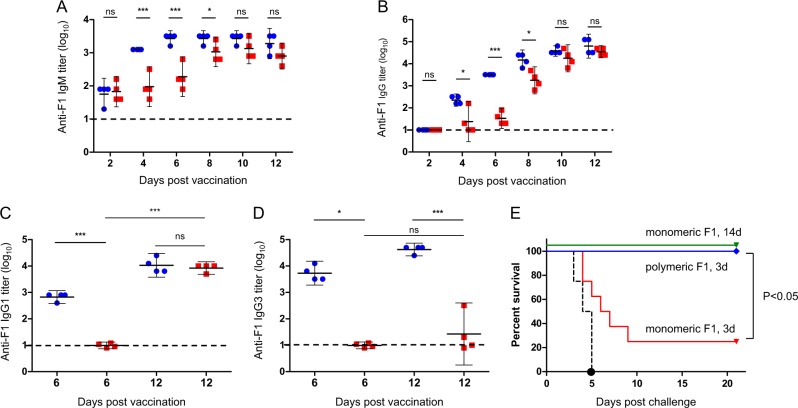
Rapid anti-plague immunity is induced by polymeric, but not monomeric, F1. Anti-F1 IgM **a**, Anti-F1 IgG **b**, Anti-F1 IgG1 **c**, and anti-F1 IgG3 **d** titers in the sera of C57BL/6J mice vaccinated with polymeric (20 μg/mouse, blue circles) or monomeric (20 μg/mouse, red squares) F1 at the indicated day post vaccination (*n* = 4 for all time points in both groups). Horizontal dashed line indicates the limit of detection. Short horizontal lines mark the GMTs ( ± 95% CI). (*P < 0.05, ***P < 0.001, ns = nonsignificant). **e** Survival rates of C57BL/6J mice vaccinated with polymeric (blue line; *n* = 8) or monomeric (red line; *n* = 8) F1 or with alum (black dashed line; *n* = 8) three days prior to a lethal challenge with *Y. pestis* Kim53. Green line marks the survival rates of mice similarly vaccinated with monomeric F1 and challenged 14 days afterwards

One of the common properties shared by many antigens that generate serological responses via B1 cells is their polymeric structure that encompasses repetitive epitopes on a single molecule.^34^ It is assumed that upon specific binging to the B-cell receptors (BCRs), these repetitive epitopes promote robust clustering of membrane-anchored BCRs, which in turn, generates an activation signal that is strong enough to promote a T-cell-independent serological response.^34^ Since the capsular F1 antigen polymerizes to form high-molecular-weight polymers,^15,16^ we addressed the question of whether the polymeric structure of F1 is required for its ability to bind B1b cells and to induce rapid onset of the humoral defense response. To this end, mice were vaccinated with the labeled monomeric form of F1 generated by circular permutation of the *caf1* gene.^35^ The association between the monomeric F1 and B1b cells was examined by flow cytometry. While the binding of F1 to peritoneal B1b cells could be clearly detected in mice vaccinated with the polymeric form of F1 (Figs. 4 and 5), no such interaction was observed following vaccination with the monomeric F1 protein. In addition, as depicted in Fig. 6a, b, anti-F1 IgM and IgG titers generated on days 4, 6, and 8 after vaccination with the polymeric F1 were significantly higher than those generated by the monomeric protein. At later time points, between days 10 and 12 post vaccination, comparable anti-F1 titers were measured in the two vaccination groups (Fig. 6a, b). These results show that the poly-epitopic structure of the F1 antigen is important for the rapid generation of antibodies against F1. Notably, whereas anti-F1 IgG1 titers were similar in the mice administered with the two forms of F1 12 days post vaccination (Fig. 6c), high anti-F1 IgG3 titers, which are a hallmark for TI serological responses,^36^ were detected only in the sera of mice vaccinated with polymeric F1 (Fig. 6d).

To assess the early protective activity afforded by polymeric vs. monomeric F1 protein, mice were vaccinated with an identical dose of the two F1 forms and, 3 days later, challenged as described above. While the mice immunized with polymeric F1 were fully protected, only 25% of the mice that were vaccinated with the monomeric F1 protein survived the challenge (Fig. 6e). Of note, monomeric F1 retained the ability to generate effective anti-plague immunity albeit at slower kinetics (Fig. 6).

Taken together, the data show that the polymeric nature of F1 is required for (a) binding to peritoneal B1b cells, (b) the rapid induction of anti-F1 antibodies and (c) the early generation of a protective response against bubonic plague.

## Discussion

The generation of vaccines that afford immunity shortly after their administration would be advantageous in scenarios of sudden outbreaks involving highly virulent and infectious pathogens. In addition, the rapid induction of protective immunity against bacterial infections could also be used for prophylaxis in combination with second-line antibiotics in cases of antibiotic resistance to the first-choice therapy.^7,8^ The recent outbreak of pneumonic plague on the island of Madagascar^4^ and the concern regarding the appearance of antibiotic-resistant *Y. pestis* strains further emphasize the benefits of the development of such countermeasures.

We recently showed the rapid induction of anti-plague protective mechanisms evoked by the live vaccine strain *Y. pestis* EV76.^37^ In addition, the F1-expressing live attenuated *Y. pseudotuberculosis* vaccine strain with the ability to induce anti-F1 titers shortly after oral immunization was recently reported.^38^ We have demonstrated that vaccination with the purified recombinant F1 capsular protein of *Y. pestis* can induce anti-plague immunity very rapidly, even when administered several hours after a lethal challenge.^30^ In the present study, we further characterized the mechanisms that afford the rapid elicitation of protective immunity against bubonic plague following vaccination with the F1 antigen. We provide evidence that the F1 antigen can induce T -independent (TI) immunity in addition to the classical T-dependent response. The TI response elicited rapid and specific IgM antibody titers against F1 in mice lacking T-helper cells (Fig. 1b). Notably, class-switching was observed as high anti-F1 IgG titers were measured in the sera of F1-vaccinated ΔCD4 mice albeit at slower kinetics (Fig. 1c). Indeed, the ability of other T-cell subsets to support antibody class-switching in the absence of T-helper cells was previously reported.^39^ As with F1, additional protective antigens are known to elicit rapid TI serological responses. These include mainly polysaccharide-based vaccines such as the pneumococcal polysaccharides and the Vi antigen from *Salmonella typhi* but also the H-binding protein (FhbA) of *Borrelia hermsii* and the outer membrane protein D of *Salmonella typhimurium*.^40–43^

It is well established that MZ B cells and B1 cells are involved in the early initiation of TI serological responses against bacterial protective antigens.^36,44^ The usage of splenectomized mice lacking spleen-restricted MZ B cells and B1-defective *Xid* mice allowed us to identify that the rapid anti-plague immunity elicited by F1 was mediated by B1 cells. The failure of *Xid* mice to rapidly generate circulating anti-F1 IgM antibodies following vaccination was associated with poor survival rates (Fig. 2b). The inability of B1-defective *Xid* mice to mount protective serological responses has also been previously reported for other TI antigens (i.e., polysaccharides).^33,45^ The effective anti-plague immunity developed in *Xid* mice following a prolonged F1 vaccination regime suggests that the classical slower-developing adaptive F1-mediated immune response was retained (Fig. 2). In line with this finding, the ability of *Xid* mice to mount serological responses against T-dependent antigens was previously reported.^46,47^

The identification of peritoneal B1b cells during the development of F1-mediated rapid immunity was further demonstrated by the direct binding of these cells to F1 protein shortly after vaccination (Fig. 5). To the best of our knowledge, this is the first report showing that the capsular F1 antigen of *Y. pestis* is recognized by B1b cells, in accordance with the role of B1b cells in generation of protective serological responses following vaccination with TI antigens.^36^

The exceptional rapid binding kinetics could explain the early induction of specific serological responses observed following vaccination with F1 (Fig. 6 and ref. ^30^), accompanied by the slower development of additional adaptive immune responses as indicated by the association of F1 with follicular B2 cells.

The natural polymeric structure of F1 was essential for the establishment of an early protective state, as monomerization of this protein by circular permutation of the *caf1*^35^ gene abolished B1b binding, delayed the specific serological response in vaccinated animals and, consequently, was ineffective in eliciting rapid defense against infection (Fig. 6). The ability of polymeric antigens to effectively activate B1b cells could be explained through clustering of membrane-bound BCRs by repetitive epitopes of the antigen.^48^ Such clustering may trigger signaling cascades that enable B1b cells to undergo clonal expansion and differentiation into antibody-secreting cells without the assistance of T cells.^36,48^ Notably, in the currently developed anti-plague subunit vaccine, a monomeric form of F1 antigen is fused to the second protective antigen, LcrV.^21^ While the F1-LcrV vaccine was found to be highly protective in prolonged immunization regimes, its ability to elicit the rapid onset of immunity should be evaluated.

We have previously documented the rapid mounting of anti-plague immunity following vaccination with a mixture of F1 and LcrV antigens^30^ strongly supporting the notion that the inclusion of LcrV in the vaccine formulation does not affect the B1b cell-mediated rapid protective response. Studies currently underway in our laboratory will assess the ability of F1-based vaccination to promote rapid protection against the pneumonic manifestation of plague, as well as the contribution of the B1b cell-mediated activation in the context of immunity against lung infection by *Y. pestis*.

Elicitation of rapid immune responses may be beneficial during infectious disease outbreaks, by enabling extension of the time window necessary for the development of classical adaptive immune responses or by providing additional countermeasures against antibiotic-resistant pathogens. Deciphering the mechanisms that afford such rapid immunity, as demonstrated in this study for the capsular F1 antigen of *Y. pestis*, could assist in improvement of other preexisting vaccines that are currently used only for prolonged immunization regimes.

## Materials and methods

### Ethics statement

This study was carried out in strict accordance with the recommendations for the Care and Use of Laboratory Animals of the National Institute of Health. All animal experiments were performed in accordance with Israeli law and were approved by the Ethics Committee for animal experiments at the Israel Institute for Biological Research (Permit Numbers: IACUC-IIBR M20-14, IACUC-IIBR M31-15, IACUC-IIBR M74-16, IACUC-IIBR M21-16, IACUC-IIBR M28-17). During the experiments, the mice were monitored daily. Humane endpoints were used in our survival studies. Mice exhibiting loss of the righting reflex were euthanized by cervical dislocation.

### Animals

Six to 8-week-old inbred C67BL/6J, ΔCD4 T cells (ΔCD4), CBA-CaJ and CBA-NJ (*Xid*) mice were purchased from Jackson Laboratories (ME, USA).

### Animal studies

#### Immunization of mice with F1

Recombinant polymeric F1 protein was purified from the culture supernatant of *Escherichia coli* cells expressing the *caf1* operon of *Y. pestis* Kimberley53 strain, as previously described.^17^ Endotoxins (including *E. coli* derived lipopolysaccharides) were removed from the purified protein preparations by the Triton X-114 phase-separation method.^49^ The residual endotoxin levels determined by LAL assay (CAMBREX) were below 1 international endotoxin unit (IEU) per 80 μg protein, clearly establishing that all observed biological activities of the preparation may be attributed solely to its F1 content. Monomerized F1 (cpCaf1) was a kind gift from Dr. Daniel Peters (Newcastle university, UK). Protein quantity was measured using the bincinconinic acid method (Pierce™ BCA Protein Assay Kit, Thermo). Polymeric and monomeric F1 proteins were resolved on gradient 4–12% polyacrylamide gel (NuPAGE, Thermo) with or without boiling. Resolved proteins were visualized with water-based Coomassie blue stain (SeeBand Forte, GeBA) (Supplementary Fig. S1 A) or transferred to a nitrocellulose membrane and blotted with in-house rabbit anti-F1 antibodies (diluted 1:250,000,^30^) (Supplementary Fig. S1 B). The SDS-PAGE pictures of polymeric and monomeric F1 proteins are depicted in Supplementary Fig. S1.

Both proteins were labeled with Alexa Fluor® 488 and 647 dyes using Molecular Probes® labeling kits according to the manufacturer instructions (Thermo Fischer, USA). The antigenicity of the polymeric labeled F1 and its ability to confer rapid onset of protection were verified. Twenty to 80 micrograms of the purified or fluorophore-labeled antigens was adsorbed to aluminum-hydroxide gel (Brennentag Biosector, Denmark) to a final concentration of 0.36%. Individual mice were immunized subcutaneously using a constant volume of 200 µl divided equally between two sites of the upper or the lower back. Mice were tail-bled, and individual IgM and IgG titers generated against F1 were determined by ELISA as previously reported.^30^ Due to limitations on the volume and frequency of bleeding of a single animal according to the recommendations for the Care and Use of Laboratory Animals, for evaluation of the timeline of antibody titers development following vaccination/challenge, each mouse was bled on intermittent time points only. IgG1 and IgG3 titers were measured using isotype specific alkaline phosphatase-conjugated goat anti-mouse antibodies (Jackson Laboratories, USA). Values are presented as log_10_ of the reciprocal of the dilution of the sample required to reach the endpoint of the test (twofold of the background absorbance at 405 nm). All experiments reported were performed with female mice. Of note, the major observations regarding the elicitation of rapid protective immunity upon polymeric F1 vaccination were confirmed in male mice (not shown).

### Mouse infection

The fully virulent *Y. pestis* strain Kimberley53 (Kim53^50^) was grown on brain-heart infused (BHI) agar plates at 28 °C. Several isolated and typical colonies were suspended in saline to generate a bacterial suspension at an optical density (660 nm) of 0.1 (equals to 10^8^ cfu/ml). This bacterial suspension was serially diluted, and a lethal dose of 100 cfu (1 cfu = 1 LD_50_) was injected subcutaneously (s.c.) into the lower right backs of the mice. The infectious dose was verified by plating diluted bacterial suspensions onto BHI agar plates.

### Flow cytometry analysis

Mice were euthanized by cervical dislocation. The spleen and the draining inguinal lymph node were excised and mashed onto 70-µm mesh (Corning, USA) to obtain single-cell suspensions. The peritoneal cavity was washed with 5 ml of 1 × PBS with 3% fetal calf serum (Biological Industries, Israel). Cells extracted from the peritoneal cavity were incubated with anti-CD16/CD32 antibodies (eBioscience, USA) and subsequently stained with B220-FITC (53-7.3, eBioscience) and CD23-PE (B3B4, eBioscience) to identify B cells and with CD11c-PE (N418, eBioscience) and Gr-1-FITC (RB6-8C5, eBioscience) to identify dendritic cells (CD11c^+^Gr-1^-^), macrophages (CD11c^mid^Gr-1^+^) and neutrophils (CD11c^-^Gr-1^+^). In addition, cells were stained with CD3-FITC (17A2, eBioscience) and CD49b-PE (DX5, eBioscience) to identify T cells (CD3^+^DX5^−^) and natural killer (NK) cells (CD3^−^DX5^+^). Peritoneal cells were also stained with B220-Briliant Violet 510 (RA3-6B2, BioLegend), IgLk-FITC (187.1, BD Biosciences), IgD-PE/Cy7 (11-26c, eBioscience), CD23-Briliant Violet 421 (B3B4, BioLegend), and CD5-PerCP Cy5.5 (53-7.3, eBioscience). PerCP Cy5.5 isotype control antibodies (eBR2a, eBioscience) were used to validate the specificity of the CD5 staining. Cells were analyzed using a FACSCalibur flow cytometer or a FACSAriaIII cell sorter (BD biosciences) and FlowJo software (Three Star).

### Statistical analysis

Differences between survival curves were assessed using a Kaplan–Meier analysis with a log-rank (Mental-Cox) test, provided in the GraphPad Prism 5 statistical pack. Differences of interest between titer endpoints (represented as log_10_ of the reciprocal of the dilution of the sample required to reach the endpoint of the test (twofold of the background absorbance at 405 nm)) were compared using independent-samples two-sided *t*-tests. Differences were considered statistically significant at *P* < 0.05.

## Electronic supplementary material

Levy et al Supplementary information

## Data Availability

The datasets generated during and/or analyzed during the current study are available from the corresponding author on reasonable request.

## Electronic supplementary material

**Supplementary information** accompanies the paper on the *npj Vaccines* website (10.1038/s41541-018-0087-z).
