# Supplementary material for: Targeting of the Yersinia pestis F1 capsular antigen by innate-like B1b cells mediates a rapid protective response against bubonic plague
Source: NPJ Vaccines. 2018 Oct 19;3:52. doi: 10.1038/s41541-018-0087-z (PMC6195588; doi:10.1038/s41541-018-0087-z)
Supplement: Supplementary file 1 — Levy et al Supplementary information [file 41541_2018_87_MOESM1_ESM.pdf]

Figure S1

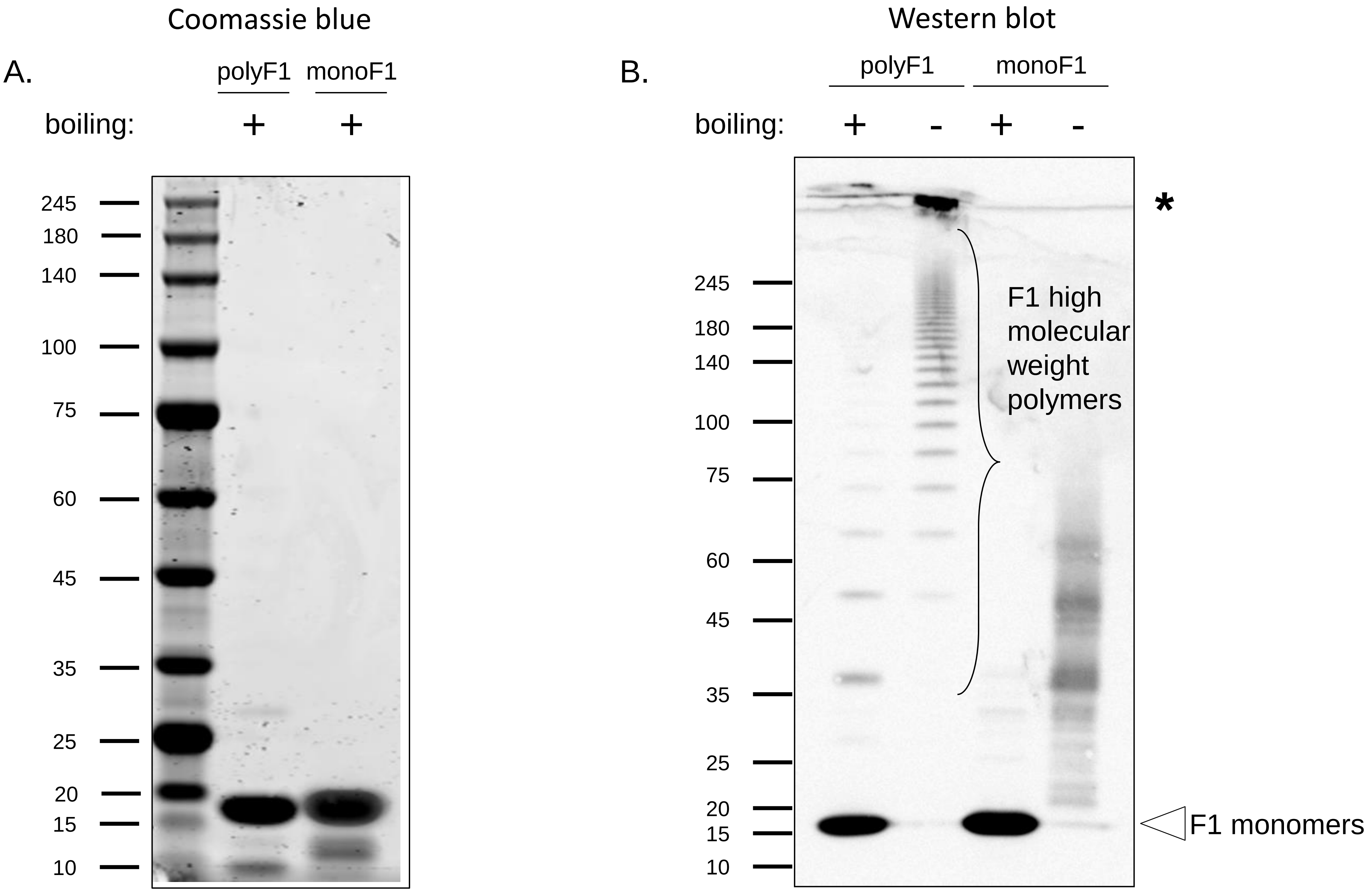

Coomassie blue
A.
polyF1
monoF1
boiling:
+
+
245
180
140
100
75
60
45
35
25
20
15
10
Western blot
B.
polyF1
monoF1
boiling:
+
-
+
-
*
245
180
140
100
75
60
45
35
25
20
15
10
F1 high molecular weight polymers
F1 monomers

Figure S2

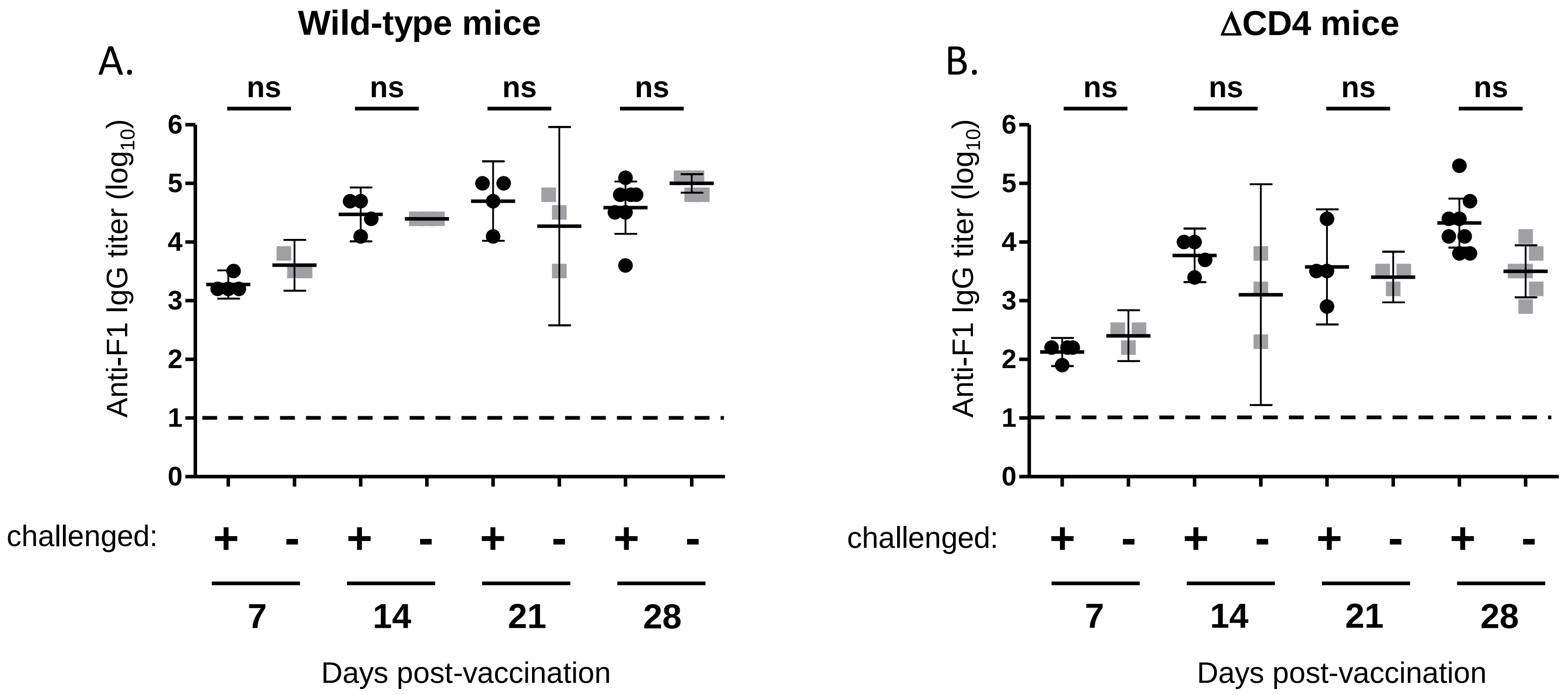

Wild-type mice
A.
ns
ns
ns
ns
Anti-F1 IgG titer (log10)
6
5
4
3
2
1
0
challenged:
+ - + - + - + -
7
14
21
28
Days post-vaccination
ΔCD4 mice
B.
ns
ns
ns
ns
Anti-F1 IgG titer (log10)
6
5
4
3
2
1
0
challenged:
+ - + - + - + -
7
14
21
28
Days post-vaccination

Figure S3

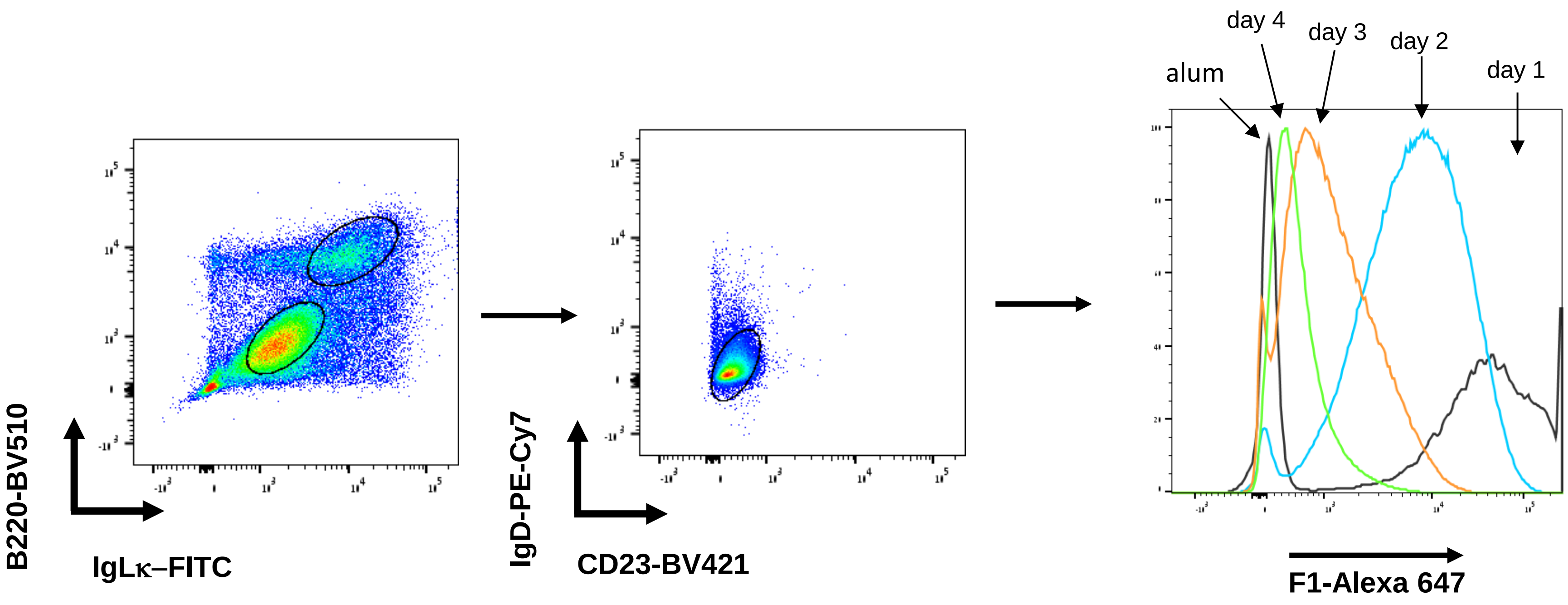

B220-BV510
IgLκ–FITC
IgD-PE-Cy7
CD23-BV421
alum
day 4
day 3
day 2
day 1
F1-Alexa 647

Figure S1. SDS-PAGE analyses of polymeric F1 and monomeric F1.

A) Coomassie blue staining of equal amounts of polymeric F1 (polyF1) and monomeric F1 (monoF1) resolved by 4-16% SDS-PAGE after boiling. B) Western blot analysis using rabbit anti-F1 antibodies (diluted 1:250,000) of polyF1 and monoF1 resolved by 4-12% SDS-PAGE with or without boiling. F1 monomers and F1 high-molecular weight polymers are indicated. Without boiling the majority of polyF1 does not resolve in the gel (marked by an asterisk). Molecular weight markers in kilodalton are shown on the left. The Coomassie blue stained gel and the blot derives from the same experiment and were processed in parallel.

Figure S2. Anti-F1 IgG titer development in vaccinated mice, with or without a lethal challenge.

Anti-F1 IgG titers measured in the sera of wild-type C57BL/6J mice (A) and ΔCD4 mice (B) at the indicated day post vaccination with 80μg/mouse polymeric F1 alone (gray squares, n=3 for each of the time points in both mice strain groups, except for day 28 on which n=6 for both groups), or also following a subcutaneous challenge with $100LD_{50}$ of *Y. pestis* Kim53 on day 7 post-vaccination (black circles, n=4 for each of the time points in both mice strain groups, except for day 28 on which n=7 and n=8 in the wild-type and ΔCD4 groups, respectively). Short horizontal lines mark the GMT (±95%CI). Horizontal dashed line indicates the limit of detection. (ns = nonsignificant).

Figure S3. Kinetics of F1 association with peritoneal B1b cells.

Representative dot plot and histogram analyses of gated cells extracted from the peritoneal cavity of C57BL/6 mice vaccinated with the adjuvant (n=3) or with the labeled F1 (n=3). The black line marks the staining of F1 associated with a mixture (1:1 ratio) of $B220^{mid}IgLk^{mid}IgD^{-}CD23^{-}$ (B1) cells extracted from control or from F1-vaccinated mice, one day post vaccination. Left peak marks negative staining (alum), and right peak marks positive staining at this time point. Magenta, orange and green lines mark F1 staining in $B220^{mid}IgLk^{mid}IgD^{-}CD23^{-}$ (B1) cells extracted from F1-vaccinated mice at two, three and four days post vaccination, respectively.
